# (Not) far from home: No sex bias in dispersal, but limited genetic patch size, in an endangered species, the Spotted Turtle (*Clemmys guttata*)

**DOI:** 10.1002/ece3.9734

**Published:** 2023-01-04

**Authors:** Eric B. Liebgold, Myra J. Dickey, Stephanie M. Lamb, Hunter J. Howell, Tami S. Ransom

**Affiliations:** ^1^ Department of Biological Sciences Salisbury University Salisbury Maryland USA; ^2^ Department of Entomology Texas A & M University College Station Texas USA; ^3^ Department of Biology University of Miami Coral Gables Florida USA; ^4^ Department of Environmental Studies Salisbury University Salisbury Maryland USA

**Keywords:** endangered species, fine‐scale genetic spatial structure, freshwater turtle, microsatellite loci, population genetics, sex‐biased dispersal

## Abstract

Sex‐biased dispersal is common in many animals, with male‐biased dispersal often found in studies of mammals and reptiles, including interpretations of spatial genetic structure, ostensibly as a result of male–male competition and a lack of male parental care. Few studies of sex‐biased dispersal have been conducted in turtles, but a handful of studies, in saltwater turtles and in terrestrial turtles, have detected male‐biased dispersal as expected. We tested for sex‐biased dispersal in the endangered freshwater turtle, the spotted turtle (*Clemmys guttata*) by investigating fine‐scale genetic spatial structure of males and females. We found significant spatial genetic structure in both sexes, but the patterns mimicked each other. Both males and females typically had higher than expected relatedness at distances <25 km, and in many distance classes greater than 25 km, less than expected relatedness. Similar patterns were apparent whether we used only loci in Hardy–Weinberg equilibrium (*n* = 7) or also included loci with potential null alleles (*n* = 5). We conclude that, contrary to expectations, sex‐biased dispersal is not occurring in this species, possibly related to the reverse sexual dimorphism in this species, with females having brighter colors. We did, however, detect significant spatial genetic structure in males and females, separate and combined, showing philopatry within a genetic patch size of <25 km in *C. guttata*, which is concerning for an endangered species whose populations are often separated by distances greater than the genetic patch size.

## Graphical abstract

Sex‐biased dispersal is common in many animals, with male‐biased dispersal often found. We found significant spatial genetic structure in both sexes, but male and female dispersal patterns mimicked each other, with higher than expected related at distances <25 km. Lack of male‐biased dispersal was surprising, but a genetic patch size of <25 km is concerning for an endangered species whose populations are often separated by large distances.

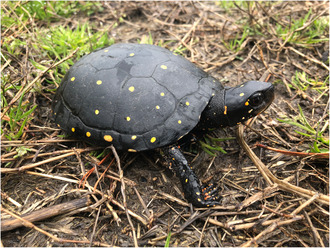

## 1 INTRODUCTION

Sex‐biased dispersal occurs when one sex is more likely to disperse or tends to disperse further than the other sex (Greenwood, 1980). The sex that disperses less is more likely to remain philopatric to its natal area (Greenwood, 1980). Sex‐biased dispersal has only been widely studied in birds (reviewed in Greenwood, 1980; Pusey, 1987) and mammals (reviewed in Greenwood, 1980; Lawson Handley & Perrin, 2007) but has been detected in many other vertebrate taxa (amphibians: e.g., Liebgold et al., 2011; Palo et al., 2004; teleost fish: e.g., Bekkevold et al., 2004, squamates: e.g., Dubey et al., 2008; Stow et al., 2001; and turtles: e.g., Moore et al., 2020; Paquette et al., 2010; Sheridan et al., 2010). While inbreeding avoidance is considered an ultimate cause of sex‐biased dispersal, it does not help predict which sex is more likely to disperse or disperse further (Prugnolle & De Meeus, 2002; Pusey, 1987). This is because dispersal of either sex alleviates the potential for inbreeding (Lebigre et al., 2010; Perrin & Mazalov, 2000). However, there are a number of ecologically‐based hypotheses that have been proposed based on bird and mammal systems for why a particular sex is the dispersing sex (Goudet et al., 2002). Despite their formation based on data from birds and mammals, these hypotheses, along with relevant ecological information, can help us predict which sex is more likely to disperse in non‐avian and non‐mammalian taxa (e.g., Dubey et al., 2008; Liebgold et al., 2011).

Many bird species exhibit female‐biased dispersal (Prugnolle & De Meeus, 2002; Pusey, 1987) as a result of resource competition (Greenwood, 1980). In birds, males are often involved in parental care, most typically feeding offspring, so males will remain philopatric to retain familiarity with their local environment, which is associated with foraging success and increased survival of offspring (Greenwood, 1980; Perrin & Mazalov, 1999). In contrast, in mammals, males are more likely to disperse to avoid kin competition (Greenwood, 1980), potentially due to male–male competition for mates and resulting avoidance of kin competition (Greenwood, 1980; Lawson Handley & Perrin, 2007). Additionally, female mammals provide most parental investment (Greenwood, 1980; Pusey, 1987) and may benefit more from philopatry (and familiarity with the local foraging environment) than males (Perrin & Mazalov, 1999).

There has been less generalization about potential causes and direction of sex‐biased dispersal in non‐avian reptiles despite a plethora of studies investigating and documenting its existence. All of the studies testing for sex‐biased dispersal found male‐biased dispersal (snakes: Dubey et al., 2008; Folt et al., 2019; Francois et al., 2021; Hofmann et al., 2012; Keogh et al., 2007; Rivera et al., 2006; Zhong et al., 2017; Zwahlen et al., 2021; lizards: Dubey & Shine, 2010; Johansson et al., 2008; Olsson & Shine, 2003; Stow et al., 2001; crocodiles: Tucker et al., 1998; turtles: Casale et al., 2002; Chaloupka & Limpus, 2002; FitzSimmons et al., 1997; Lee et al., 2007; Moore et al., 2020; Paquette et al., 2010; Sheridan et al., 2010) with the exception of one species of lizard (Chapple & Keogh, 2005). Our understanding of which sex is more likely to disperse during sex‐biased dispersal in birds and mammals can provide insight into the causation underlying the general pattern of male‐biased dispersal in non‐avian reptiles. Similar to most mammals, many reptiles have intense male–male competition and polygynous mating systems, which may lead to male‐biased dispersal (Rivera et al., 2006; Tucker et al., 1998). Non‐avian reptiles frequently use male–male combat in territorial interactions or to compete for mates (reviewed in Vitt & Caldwell, 2014). In accordance with the local mate competition hypothesis (Greenwood, 1980), male non‐avian reptiles may disperse to avoid kin competition (Dubey et al., 2008), as has been observed in many species of mammals (Lawson Handley & Perrin, 2007). Unlike avian reptiles (i.e., birds), males in non‐avian reptile species tend to have little to no parental investment or parental care (reviewed in Vitt & Caldwell, 2014).

Few studies have tested for sex‐biased dispersal in turtles (clade Testudines), however, and all such studies have been either in marine turtles (e.g., Casale et al., 2002; Lee et al., 2007; Sheridan et al., 2010) or fully‐terrestrial turtles (Moore et al., 2020; Paquette et al., 2010), with no tests of sex‐biased dispersal in freshwater turtle species to date. All turtles studied so far displayed male‐biased dispersal. This is potentially because, similar to other reptiles such as squamates, turtles have little‐to‐no male parental investment and, in many species, male–male competition is common (Vitt & Caldwell, 2014). Therefore, turtles, like other non‐avian reptiles, may be more likely to display male‐biased dispersal due to avoidance of male–male competition with kin, consistent with the local mate competition hypothesis (sensu Greenwood, 1980). Alternatively, male‐biased dispersal may be present because it is an ancestral trait of all turtles or all reptiles.

Like most species of turtles, and all freshwater turtles, nothing is known about sex‐biased dispersal in spotted turtles (*Clemmys guttata*), and little is known about sex‐biased dispersal in other turtles in the family Emydidae (but see Moore et al., 2020; Sheridan et al., 2010). *Clemmys guttata* is found in the eastern part of North America and occupies a variety of wetland habitats that are often semi‐terrestrial although they aggregate in shallow ponds to breed (Ernst & Lovich, 2009). *Clemmys guttata* is listed on the IUCN Red List as endangered and is listed in Appendix II of CITES (Convention on International Trade in Endangered Species; CITES, 2013) as well as currently being reviewed by the U.S. Fish and Wildlife Service to determine whether it should be considered under the Endangered Species Act. *Clemmys guttata* has experienced 50% reduction in numbers of individuals in three generations (Van Dijk, 2011) and studies have shown that many populations of *C. guttata* have low densities, which has led to concerns about maintenance of genetic diversity and calls for the need for increased genetic conservation (Ernst & Lovich, 2009).

If a population is small, it has a higher chance of inbreeding and, once inbreeding starts to occur, the population size is likely to continue to get smaller each generation unless new individuals or alleles are introduced, which can be alleviated by sex‐biased dispersal (Guillaume & Perrin, 2006; e.g., Folt et al., 2019). Maintaining genetic diversity, potentially in part via dispersal, is critical to endangered turtle species like *C. guttata* so they can avoid negative effects of inbreeding depression (Parker & Whiteman, 1993). Inbreeding can increase homozygosity within a population (Charlesworth & Charlesworth, 1987), potentially leading to decreased fitness (inbreeding depression) and smaller overall population size (Charlesworth & Charlesworth, 1999).

Few studies on genetic variation in pond turtle (Family Emydidae) populations have been done and very few on turtle species of conservation concern to understand the potential threats from inbreeding and the potential benefits of sex biases in dispersal. Blanding's turtle (*Emydoidea blandingii*), and bog turtles (*Glyptemys muhlenbergii*) turtles are both threatened or at risk throughout their range with populations of *G. muhlenbergii* having low genetic diversity, ostensibly due to habitat loss and small population sizes (Rosenbaum et al., 2007). In *E. blandingii*, however, genetic diversity is high because they seem to have sufficient habitats to allow gene flow (McCluskey et al., 2016, 2022; Reid et al., 2016) and are long‐lived (McCluskey et al., 2016), which alleviates or delays the signature of genetic isolation (McCluskey et al., 2016) despite some potential ecological constraints to long‐distance movements (McCluskey et al., 2022). The first genetic study on *C. guttata* in the northern extent of its range in Canada found population differentiation (Davy et al., 2014). In contrast, further toward the center of their range in Rhode Island, Buchanan et al. (2019) recently reported little genetic differentiation between populations and found similar genetic diversity to a common species, the Eastern Painted Turtle (*Chrysemys picta*).

We hypothesized that the *C. guttata* will exhibit male‐biased dispersal, ostensibly to avoid inbreeding (Lebigre et al., 2010; Perrin & Mazalov, 2000), similar to other turtles and other species of reptiles. Male‐biased dispersal was found in nearly every other species of reptile studied. While this could be due to fixation of male‐biased dispersal from the ancestor of all reptiles, in most of the reptiles studied, male reptiles often display more intense intra‐specific competition, potentially leading them to be more likely to have males disperse to avoid kin competition (e.g., Perrin & Mazalov, 2000).

## 2 METHODS

### 2.1 Study species

*Clemmys guttata* is the only extant member of the genus *Clemmys*, and ranges from Maine to central Florida (Ernst & Lovich, 2009). Female *C. guttata* are not sexually active until they are 12 years old (Enneson & Litzgus, 2009; Litzgus, 2006). Mating occurs in freshwater vernal pools or roadside ditches (reviewed in Ernst & Lovich, 2009). Once mating has occurred, a female will lay a small clutch (three to five eggs) nearby the body of water (Ernst & Lovich, 2009). Individuals of *C. guttata* are extremely long‐lived and can potentially live to be 110 years old (Litzgus, 2006; Seburn, 2018). Little is known about dispersal in *C. guttata* (reviewed in Ernst & Lovich, 2009).

### 2.2 Study sites

We studied *C. guttata* at five sites in Maryland and Delaware located on the coastal plain of the Delmarva Peninsula and the western side of the Chesapeake Bay. The sites were separated by variable distances (31.2–175.0 km, Table 1, Figure S1). Due to concerns about human collection for the pet trade (CITES, 2013), MD Department of Natural Resources and DE Department of Natural Resources and Environmental Control have prudently prohibited publication of spatial information or maps pertaining to *C. guttata* locations (sensu Lindenmayer & Scheele, 2017). All of these *C. guttata* sites (Table 1) can be characterized as isolated freshwater wetlands based on their geographic location (sensu Tiner, 2003), with *C. guttata* present in one or two ponds at a site (sites A, C, and E) or in multiple wetlands at a site (sites B and D, which were divided into three or two subsites, respectively; see Figure S1). Wetlands located within this region have no geographic interactions with other wetlands (Tiner, 2003). But while these wetlands are not contiguous and landscapes between wetlands are not suitable habitat for *C. guttata*, the connective landscapes are potentially permeable to *C. guttata* and other turtle movements to some degree (Lamb, 2018). Each site contains breeding habitat for *C. guttata* in the form of vernal pools and/or roadside ditches, which typically fill in the wet season and then dry out in most years and forested terrestrial habitat occupied in the non‐breeding season (reviewed in Ernst & Lovich, 2009). *Clemmys guttata* captured within most sites were separated by <1 km. However, one site (site D) had three near, but separate, subsites with bodies of water that provided suitable breeding habitat with their centers separated by <8 km. However, the subsites were not fragmented habitats but were connected by forests and marshes that we were unable to survey. Likewise, site B had two subsites. The center of subsite B2 was 17 km to the west of center of the main area (B1). Site B probably represents a single population as the edges of the habitat of the subsites were separated only by <8 km through mostly forested landscapes (albeit on private lands where we could not sample).

**TABLE 1:** Pairwise geographical distances (km) among (a) five *Clemmys guttata* sites on and near the Delmarva Peninsula in Maryland and Delaware and (b) subsites at sites B and D

(a)	Site A	Site B	Site C	Site D	Site E
Site A	‐	46.9	61.9	85.3	175.0
Site B	‐	‐	79.1	84.7	145.4
Site C	‐	‐	‐	31.2	142.4
Site D	‐	‐	‐	‐	110.1
Site E	‐	‐	‐	‐	‐

(b)	B1	B2	D1	D2	D3
B1	‐	16.9	‐	‐	‐
B2	‐	‐	‐	‐	‐
D1	‐	‐	‐	3.9	7.4
D2	‐	‐	‐	‐	3.7
D3	‐	‐	‐	‐	‐

### 2.3 Tissue collection

**FIGURE 1:**
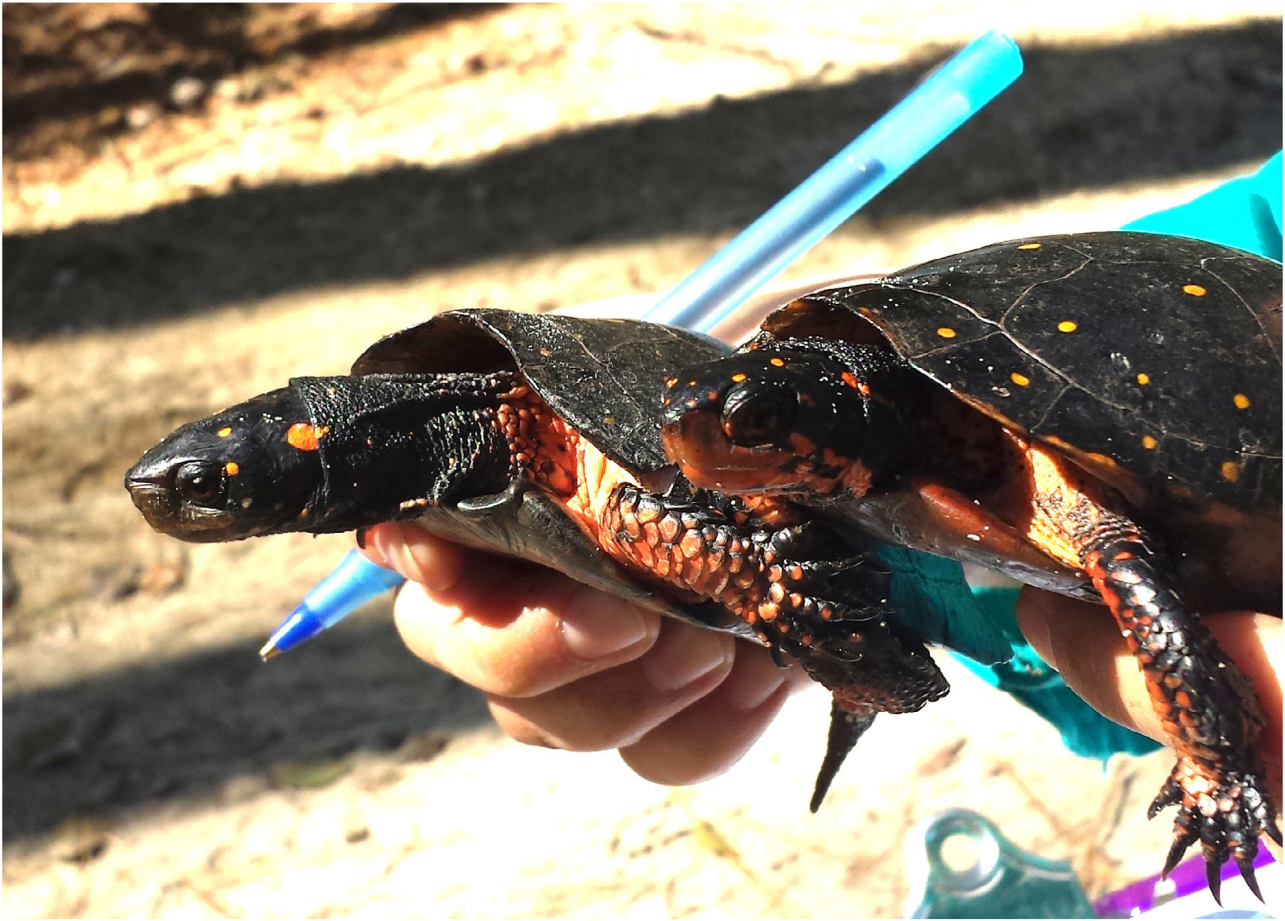
*Clemmys guttata* sexual dimorphism, with females (right) having orange chins and orange eyes, while males (left) have dark chins and brown eyes (Photo by Eric B. Liebgold)

During the months of January–July in 2016, each site was visited for four trapping periods and, from January to June in 2017, each site was visited for three trapping periods to mark‐recapture *C. gutatta*. We used handmade D‐hoop traps (87 × 50 cm) and collapsible mesh minnow/crawfish bait traps (61 × 30.5 cm; Promar) baited with sardines in oil. Once traps were set, they were checked every 24 h. When turtles were captured, we identified spatial coordinates of the trap using GPS units (Garmin), determined their sex (Figure 1) and notched their marginal scutes in a unique pattern to identify individuals (modified from Cagle, 1939). Each *C. guttata* adult was swabbed in their oral cavity to collect saliva and cheek cells for DNA analysis (sensu Poschadel & Möller, 2004). Swabs were then processed by air‐drying for 15 min and freezing until DNA extraction.

### 2.4 Microsatellite genotyping

**FIGURE 2:**
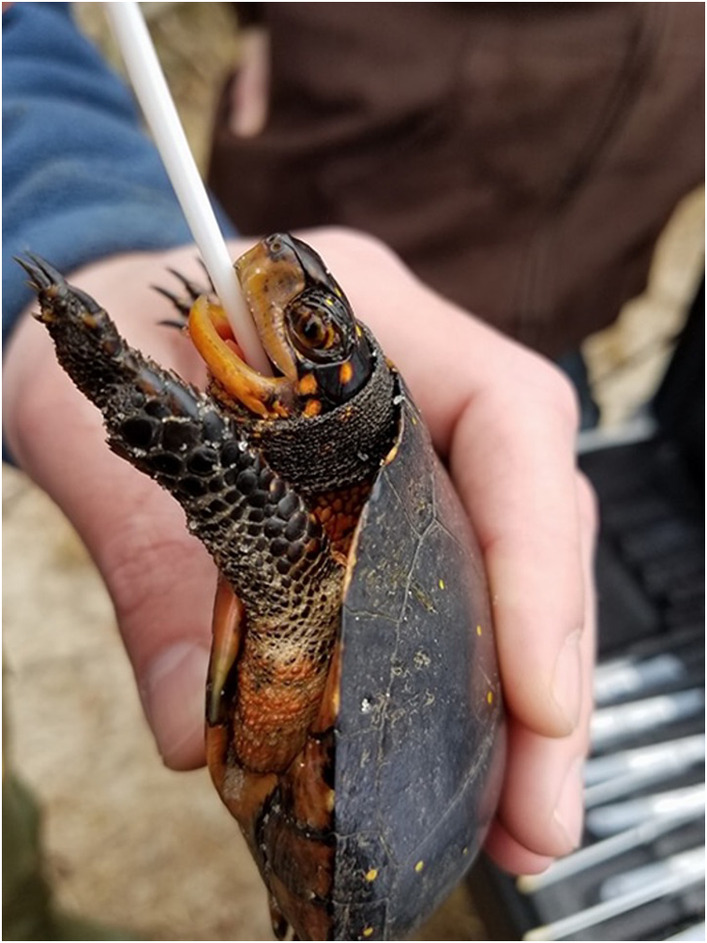
Buccal cheek cell tissue collection from *Clemmys guttata* using a buccal swab (photo by Stephanie M. Lamb)

We extracted DNA from buccal swabs Figure (2) using a MasterAm Buccal Swab DNA Extraction Kit (Epicentre). We genotyped male and female adult spotted turtles at 12 repeat microsatellite loci (Table 2), 10 that were originally isolated from bog turtles (*Glyptemys muhlenbergii*), and two microsatellite loci originally isolated from Blanding's turtles (*Emydoidea blandingii*), all of which have been previously used to amplify *C. guttata* DNA (Davy et al., 2014; King & Julian, 2004; Osentoski et al., 2002). The loci were amplified using PCR protocols modified from King and Julian (2004) and McCluskey et al. (2016). PCR products were visualized using an ABI 3730xl Automated Sequencer (Applied Biosystems). Alleles were sized and scored using GENEMARKER (v2.6, Softgenetics). We tested for Hardy–Weinberg equilibrium and heterozygote deficiency at each locus at each site, and linkage disequilibrium for each pair of loci using GENEPOP (v4.0.10) at 1000 iterations (Raymond & Rousset, 1995; Rousset, 2008). We used MICROCHECKER (v2.2.3) to check each locus for potential null alleles and scoring errors (Oosterhout et al., 2004). We calculated observed and expected heterozygosities (*H*_O_ and *H*_E_, respectively) and number of alleles per locus (Table 2) and *H*_O_, *H*_E_ and the number of rarified alleles per locus per population (sensu Kalinowski, 2004, 2005; Table 3). We then used the linkage disequilibrium method (LDNe) implemented in N_e_ESTIMATOR (v2.01, Do et al., 2014) to estimate effective population size (*N*_e_) for each site and compared *N*_e_ to mark‐recapture estimates of population size (*N*_c_; sensu Frankham, 1995; Nunney, 1995), calculated with the POPAN estimator in Program MARK (Cooch & White, 2005; White & Burnham, 1999) in Lamb (2018).

**TABLE 2:** Variation in the 12 microsatellites used: Range of alleles, number of alleles, observed heterozygosity (*H*_O_) and expected heterozygosity (*H*_E_) for each locus

Locus	Size range (base pairs)	Number of alleles	*H*_O_	*H*_E_
*EB09*	***113–167***	***22***	***0.250***	***0.892***
*EB19*	***93–99***	***3***	***0.194***	***0.513***
GmuA19	123–135	7	0.664	0.670
GmuB08	188–209	7	0.492	0.631
GmuD16	122–188	15	0.758	0.865
*GmuD21*	***138–178***	***9***	***0.438***	***0.635***
*GmuD55*	***177–213***	***10***	***0.392***	***0.808***
GmuD79	157–189	8	0.618	0.779
*GmuD87*	***198–276***	***18***	***0.621***	***0.922***
GmuD88	110–134	7	0.464	0.597
GmuD114	93–109	5	0.396	0.530
GmuD121	129–162	9	0.627	0.819

*Note*: Loci with significant deviations from Hardy–Weinberg equilibrium (italicized) and significant heterozygote deficiency (bolded) at a majority of sites (after a Bonferroni correction, Table 2) are bolded and italicized.

**TABLE 3:** Variation in the 12 microsatellite loci used: Rarified number of alleles (*R*_a_), observed heterozygosity (*H*_O_) and expected heterozygosity (*H*_E_) for each locus at each site

Locus	Site A *N* = 41			Site B *N* = 49			Site C *N* = 30			Site D *N* = 25			Site E *N* = 19		
	*R*_a_	*H*_o_	*H*_E_	*R*_a_	*H*_o_	*H*_E_	*R*_a_	*H*_o_	*H*_E_	*R*_a_	*H*_o_	*H*_E_	*R*_a_	*H*_o_	*H*_E_
*Eb09*	6.2	***0.227***	***0.762***	7.9	***0.179***	***0.912***	7.7	***0.313***	***0.859***	8.8	***0.467***	***0.936***	6.3	***0.176***	***0.706***
*Eb19*	1.9	***0.111***	***0.337***	2.9	***0.289***	***0.591***	2.5	***0.115***	***0.307***	2.7	***0.000***	***0.552***	3.0	0.421	0.679
GmuA19	2.6	0.727	0.629	2.6	0.524	0.587	3.9	0.821	0.736	3.3	0.636	0.763	3.0	0.684	0.624
GmuB08	4.6	0.600	0.582	3.0	0.465	0.566	2.6	***0.379***	***0.518***	4.5	**0.565**	**0.769**	2.8	0.385	0.582
GmuD16	5.5	0.760	0.781	5.9	0.762	0.839	5.3	0.833	0.871	2.0	0.000	0.667	5.4	0.737	0.819
*GmuD21*	3.6	***0.400***	***0.614***	4.5	**0.550**	**0.788**	3.6	0.654	0.658	3.4	***0.167***	***0.525***	3.6	*0.211*	*0.440*
*GmuD55*	5.5	**0.478**	**0.738**	4.0	***0.222***	***0.856***	5.8	***0.429***	***0.714***	7.2	**0.563**	**0.839**	3.0	0.500	0.667
GmuD79	1.0	**0.652**	**0.824**	4.3	0.583	0.765	2.0	**0.632**	**0.734**	2.0	0.846	0.757	2.8	0.357	0.680
*GmuD87*	4.6	***0.438***	***0.602***	5.4	***0.405***	***0.569***	6.0	***0.500***	***0.579***	1.0	*1.000*	*0.644*	5.3	***0.444***	***0.556***
GmuD88	3.6	*0.483*	*0.805*	3.9	0.565	0.922	3.4	0.821	0.929	1.0	*0.333*	*0.333*	2.7	0.611	0.852
GmuD114	2.0	**0.176**	**0.386**	2.7	0.500	0.547	2.4	0.533	0.549	4.3	0.555	0.680	2.0	0.500	0.437
GmuD121	2.0	0.556	0.764	6.5	0.793	0.834	3.7	**0.476**	**0.676**	2.0	**0.600**	**0.846**	2.0	0.625	0.817
Averages	3.6	0.467	0.652	4.5	0.486	0.731	4.1	0.542	0.678	3.5	0.478	0.652	3.5	0.471	0.655

*Note*: Loci with significant deviations from Hardy–Weinberg equilibrium are italicized and loci with significant heterozygote deficiency at a majority of sites (after a Bonferroni correction) are bolded.

### 2.5 Statistical analysis of spatial genetic structure

We used genetic spatial autocorrelation to determine the spatial genetic structure of each sex of *C. guttata* with the program GENALEX 6.5 (Peakall & Smouse, 2006; Smouse & Peakall, 1999). This analysis utilizes binned distance classes between individuals to generate a two‐dimensional estimate of spatial genetic structure. We chose distance class bins to maximize our pairwise relatedness sample sizes within bins based on the geographic distances between ponds within sites (0.05, 0.5, & < 1 km), distances between the centers of subsites (<25 km) and distances between sites (Table 1: 50, 100, & <200 km; sensu Banks & Peakall, 2012; Smouse et al., 2008). When philopatry occurs (for a population or for a particular sex), genetically‐similar individuals are more likely found near each other, but when dispersal is random, genetically‐similar pairs of individuals are located randomly throughout a study area (Peakall et al., 2003). Testing for genetic spatial autocorrelation allowed us to test to what extent that philopatry (in km) was occurring within our study area (aka genetic patch size) and whether dispersal propensity or genetic patch size was different for males or females (Banks & Peakall, 2012).

For each pair of individuals, a genetic relatedness coefficient, *r* (‘coefficient of relatedness’), was generated. *r* was statistically compared across the entire study area (visualized with a correlogram) and between pairs in a particular distance class compared to mean *r* of randomly paired individuals in the population (Peakall et al., 2003). *r* can be either a positive (up to 1), with greater than expected genetic similarity or negative (up to −1), with less than expected genetic similarity. A positive *r* coefficient for a distance class informs us that there is positive spatial genetic structure for that distance class and represents genetically similar individuals are more likely to be found that distance apart. Whereas, when there is a negative coefficient, individuals were less likely to be genetically similar in that distance class.

We used the heterogeneity test based on ω to compare the distance class *r* comparisons across the entire study area against a null hypothesis of no spatial genetic structure (no difference between mean *r* of a distance class and the null model of random dispersal) for the entire set of samples and then separately for each sex (Smouse et al., 2008). We then tested the actual *r* values against the expected *r* values using 9999 permutations (of *r* of randomly generated pairs) for each distance class for all individuals and for each sex. After the significances of spatial genetic structure of males and females versus a null model were calculated, we used the heterogeneity test based on *ω* and T2 test to compare spatial genetic structure between males and females. As per Banks and Peakall (2012), *α* = 0.01 for the *ω* and T2 test.

## 3 RESULTS

### 3.1 Microsatellite genotyping

**FIGURE 3:**
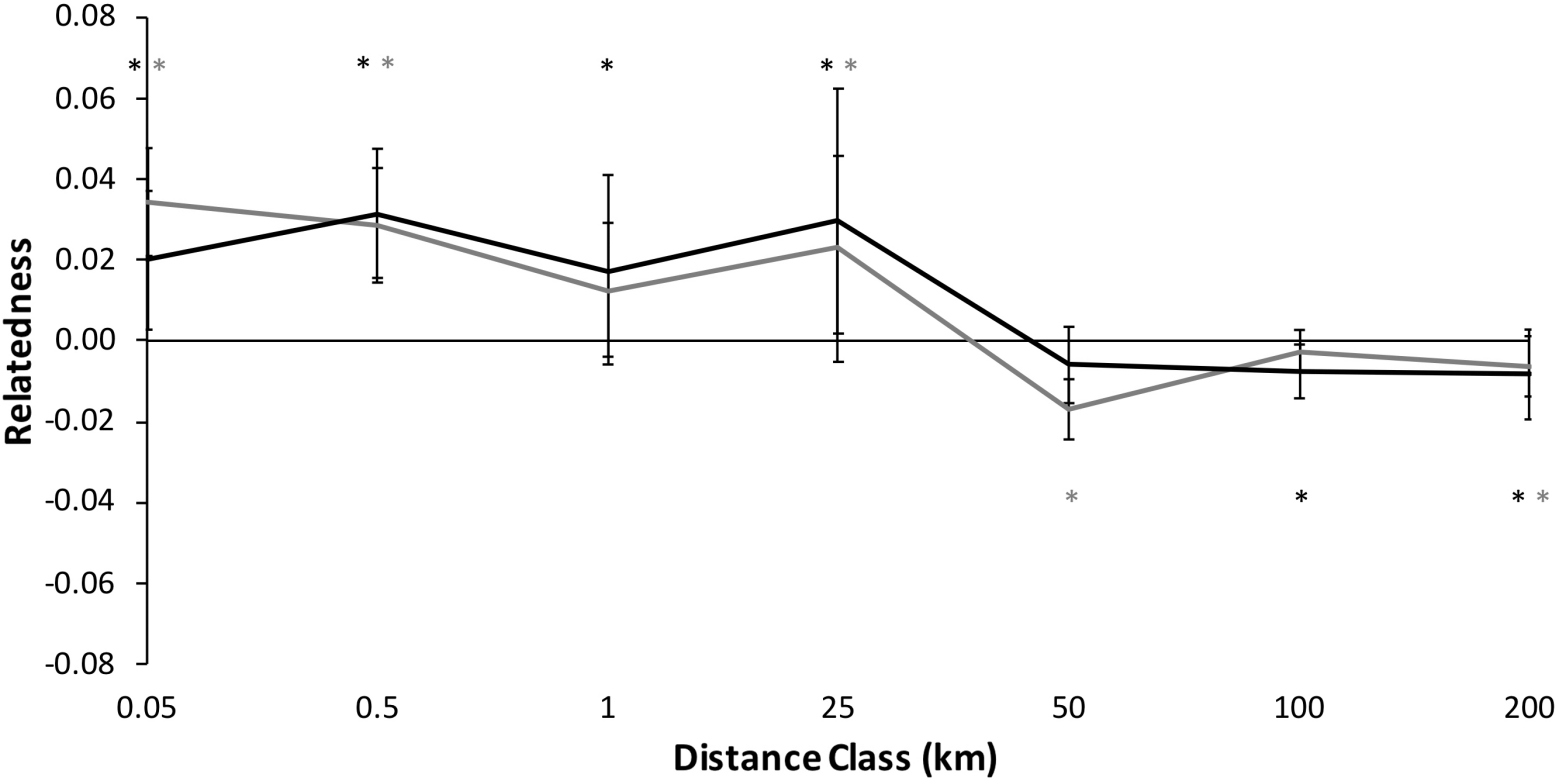
Correlogram of spatial genetic structure for male (*n* = 91; gray) and female (*n* = 75; black) *Clemmys guttata* using 12 microsatellite loci. Distance class end points are labeled on the x‐axis and error bars represent bootstrapped 95% confidence intervals. Positive numbers represent individuals that are more genetically similar than expected and negative numbers are individuals that are less genetically similar than expected (9999 permutations). */* represents that the genetic similarity within that distance class for males/females was significantly higher or lower than permutations of random individuals in the population (*α* = 0.05)

**FIGURE 4:**
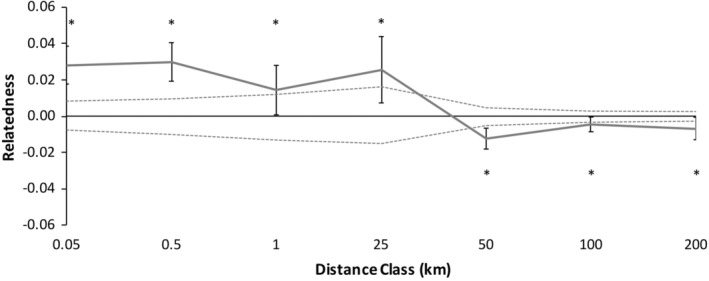
Correlogram showing combined male and female data (*n* = 164) autocorrelation across distance classes for *Clemmys guttata* using 12 microsatellite loci. Distance class end points are labeled on the x‐axis and error bars represent bootstrapped 95% confidence intervals. Positive numbers represent individuals that are more genetically similar than expected and negative numbers are individuals that are less genetically similar than expected (9999 permutations). Dashed lines are upper and lower confidence limitations under the null hypothesis that genotypes are distributed randomly. * Represents that the genetic similarity within that distance class was significantly higher or lower than permutations of random individuals in the population (*α* = 0.05)

**FIGURE 5:**
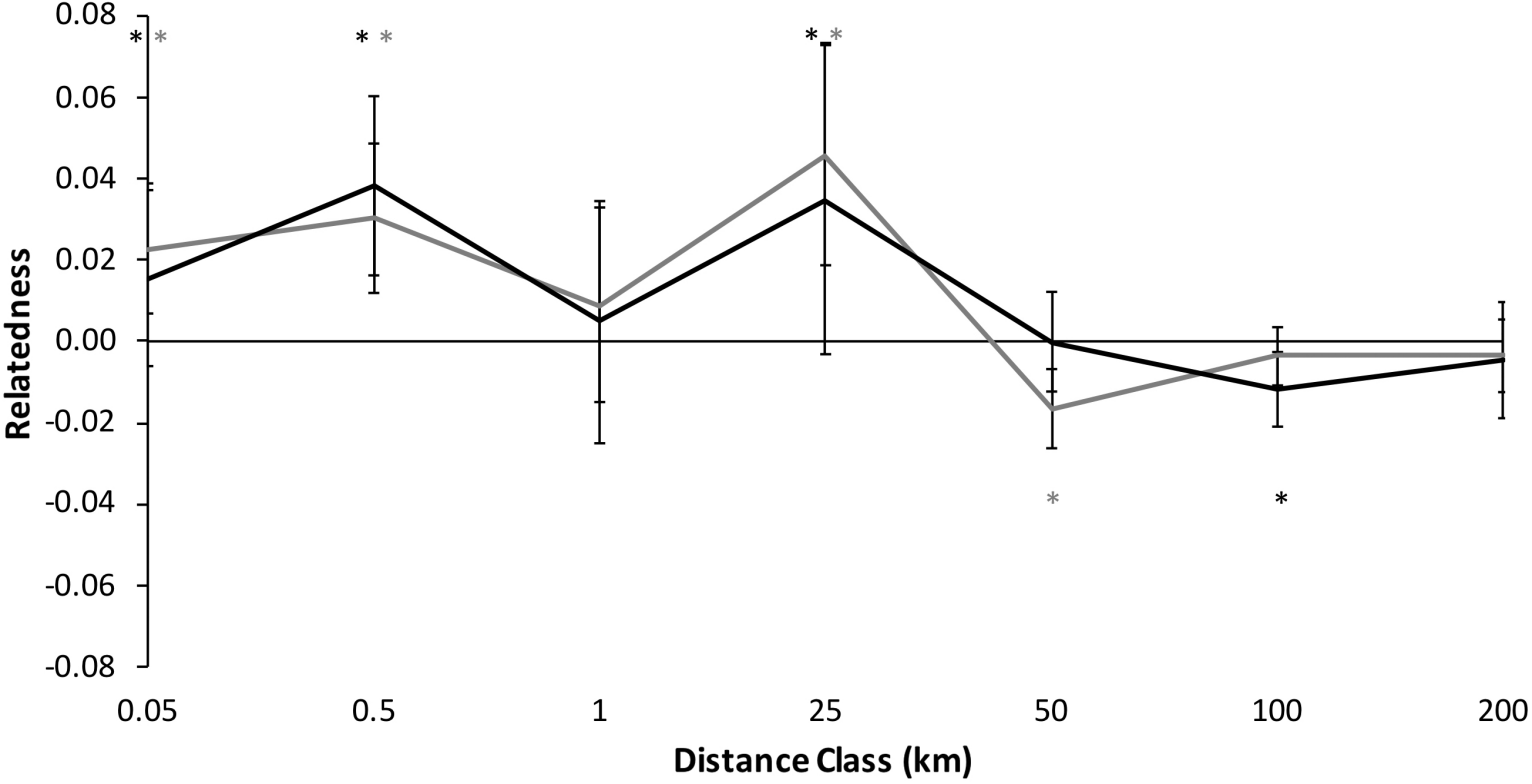
Correlogram of spatial genetic structure for male (*n* = 91; gray) and female (*n* = 75; black) *Clemmys guttata* using only the seven microsatellite loci in Hardy–Weinberg equilibrium (see text). Distance class end points are labeled on the x‐axis and error bars represent bootstrapped 95% confidence intervals. Positive numbers represent individuals that are more genetically similar than expected and negative numbers are individuals that are less genetically similar than expected (9999 permutations). */* represents that the genetic similarity within that distance class for males/females was significantly higher or lower than permutations of random individuals in the population (*α* = 0.05)

**FIGURE 6:**
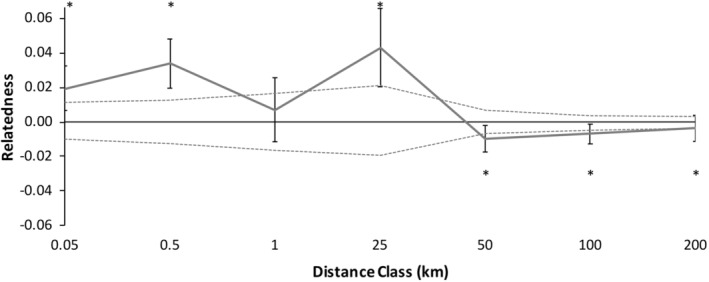
Correlogram showing combined male and female data (*n* = 75) autocorrelation across distance classes for *Clemmys guttata* using only the seven microsatellite loci in Hardy–Weinberg equilibrium (see text). Distance class end points are labeled on the x‐axis and error bars represent bootstrapped 95% confidence intervals. Positive numbers represent individuals that are more genetically similar than expected and negative numbers are individuals that are less genetically similar than expected (9999 permutations). Dashed lines are upper and lower confidence limitations under the null hypothesis that genotypes are distributed randomly. * Represents that the genetic similarity within that distance class was significantly higher or lower than permutations of random individuals in the population (*α* = 0.05)

We collected and genotyped 75 female and 91 male *Clemmys guttata*. No loci exhibited statistically significant linkage disequilibrium at any site (*p* > .05). However, while most loci at most sites were in Hardy–Weinberg equilibrium and did not have significant heterozygote deficiency (after a Bonferroni correction: *p* > .0004), we detected significant deviations from Hardy–Weinberg equilibrium and significant heterozygosity deficiency at a majority of sites (≥3) at five loci (Tables 2 & 3: Eb09, Eb19, GmuD21, GmuD87, and GmuD55). These loci at these sites deviated from Hardy–Weinberg equilibrium potentially due to null alleles (as analyzed in MICROCHECKER 2.2.3). We ran our analyses two times, once with and once without the loci that deviated from Hardy–Weinberg equilibrium at a majority of sites. Running these analyses with loci that deviated from Hardy–Weinberg equilibrium and potentially contain null alleles is potentially controversial but has been done in previous population genetic studies (Chapuis & Estoup, 2007; Séré et al., 2017). As a result, we present both sets of results and note that, when comparing spatial genetic structure results between datasets with and without these five loci, results were consistent with both types of analyses and no patterns or overall statistical significance of correlograms changed (sensu Chapuis & Estoup, 2007). Inclusion of all loci, however, did lead to minor improvement in visualization of the results and significance of statistical tests at a small number of distance classes (Table 4 vs. Table 5; Figures 3 & 4 vs. Figures 5 & 6). These increases in significance were not likely due to Type I statistical errors. Although loci with null alleles each likely have reduced power compared to loci without null alleles (and could lead to a Type II statistical error especially if they are the only loci used), increased power is expected simply by increasing the number of loci in the analysis.

**TABLE 4:** *p* values for each distance class from combined correlogram and separate male and female correlograms with 12 loci. *n* represents the number of pairwise relatedness comparisons at each distance class

Distance class (km)	0.05	0.5	1	25	50	100	200
*n* Females	263	222	109	46	603	1056	476
*p* (*r*‐random ≥ *r*‐data)	**.002**	**.0001**	**.036**	**.035**	.922	.994	.999
*p* (*r*‐random ≤ *r*‐data)	.998	1.000	.964	.965	.079	**.007**	**.001**
*n* Males	344	248	141	116	810	1488	948
*p* (*r*‐random ≥ *r*‐data)	**.0001**	**.0001**	.066	**.008**	1.000	.893	.999
*p* (*r*‐random ≤ *r*‐data)	1.000	1.000	.934	.992	**.001**	.107	**.002**
*n* Combined	607	470	250	162	1413	2544	1424
*p* (*r*‐random ≥ *r*‐data)	**.0001**	**.0001**	**.010**	**.001**	1.000	.994	1.000
*p* (*r*‐random ≤ *r*‐data)	1.000	1.000	.990	.999	**.0001**	**.006**	**.0001**

*Note*: Significant *p* values are bolded (*p* < .05).

**TABLE 5:** *p* values for each distance class from combined correlogram and separate male and female correlograms with the seven loci in Hardy–Weinberg equilibrium. *n* represents the number of pairwise relatedness comparisons at each distance class

Distance class (km)	0.05	0.5	1	25	50	100	200
*n* Females	263	222	109	46	603	1056	476
*p* (*r*‐random ≥ *r*‐data)	**0.049**	**0.001**	0.366	**0.050**	0.523	0.997	0.931
*p* (*r*‐random ≤ *r*‐data)	0.952	1.000	0.635	0.950	0.478	**0.003**	0.069
*n* Males	344	248	141	116	810	1488	948
*p* (*r*‐random ≥ *r*‐data)	**0.003**	**0.0003**	0.206	**0.0002**	1.000	0.907	0.939
*p* (*r*‐random ≤ *r*‐data)	0.998	1.000	0.794	1.000	**0.0004**	0.093	0.061
*n* Combined	607	470	250	162	1413	2544	1424
*p* (*r*‐random ≥ *r*‐data)	**0.0001**	**0.0001**	0.205	**0.0001**	0.996	0.997	0.976
*p* (*r*‐random ≤ *r*‐data)	0.999	1.000	0.798	1.000	**0.004**	**0.003**	**0.024**

*Note*: Significant *p* values are bolded (*p* < .05).

#### 3.1.1 Estimates of effective population size (*N*_e_)

Estimates of population size (*N*_c_) were always higher than estimates of *N*_e_ but *N*_e_/*N*_c_ ratios varied by site (Table 6), with the lowest ratios for the site with the smallest *N*_c_ and the site on the western side of the Chesapeake Bay, which has had severe declines (Howell et al., 2019).

**TABLE 6:** Estimates of mark‐recapture (census) population size (*N*_c_, from Lamb, 2018), effective population size (*N*_e_) and *N*_e_/*N*_c_ ratio for *Clemmys gutatta* from five sites on and near the Delmarva Peninsula in Maryland and Delaware

	*N*_c_ (95% CI)	*N*_e_	*N*_e_/*N*_c_
Site A	89 (54–134)	48.6	0.531
Site B	231 (168–386)	161.2	0.698
Site C	60 (39–273)	27.3	0.455
Site D	42 (30–119)	9.5	0.226
Site E	117 (106–165)	36.1	0.309

### 3.2 Statistical analysis of spatial genetic structure utilizing all 12 loci

#### 3.2.1 Patterns in males

Spatial genetic structure of male *C. gutatta* significantly deviated from the null model (Figure 3; *ω* = 87.76, *p* = .0001*, α* = 0.01 sensu Banks & Peakall, 2012; Smouse et al., 2008). Males were significantly more genetically similar at shorter distance classes <25 km except for a non‐significant trend to be less similar at 0.5–1 km (Figure 3; Table 4). Beyond 25 km, male *C. guttata* were significantly less genetically similar than expected at each distance class except at 50–100 km (Figure 3; Table 4).

#### 3.2.2 Patterns in females

Spatial genetic structure of female *C. gutatta* significantly deviated from the null model (Figure 3, *ω* = 72.47, *p* = .0001*, α* = 0.01). Females were significantly more likely to be genetically similar within shorter distance classes, 0–25 km (Table 4). At 25–50 km, female spotted turtles had a non‐significant trend to be less genetically similar and beyond 50 km, female spotted turtles were significantly less genetically similar than expected (Figure 3; Table 4).

#### 3.2.3 Combined patterns of males and females

We found no significant differences between males and females (Figure 3, *ω* = 12.42, *p* = .573) We found no significant differences between males and females in any distance class using the T2 test (all *p* ≥ .092). This result was consistent with males and females showing similar patterns of dispersal, with no evidence for sex‐bias dispersal.

Because there were no significant differences in spatial genetic structure between males and females, we combined the sexes data to test for overall spatial genetic structure patterns with higher spatial genetic structure resolution (Figure 4). Spatial genetic structure of the combined correlogram significantly deviated from the null model (Figure 4, *ω* = 106.70, *p* = .0001). Individuals of *C. guttata* were significantly more likely to be genetically similar within shorter distance classes, <25 km (Figure 4; Table 4). Beyond 25 km, adult spotted turtles were significantly less genetically similar at each distance class than random pairs of individuals in the study area (Figure 4; Table 4).

### 3.3 Statistical analysis of spatial genetic structure utilizing only loci in Hardy–Weinberg equilibrium

#### 3.3.1 Patterns in males

Spatial genetic structure of male *C. gutatta* significantly deviated from the null model (Figure 5, *ω* = 74.31, *p =* .0001*, α* = 0.01). Males were significantly more genetically similar within shorter distance classes 0.05–25 km, except from 0.5 to 1 km (Table 5). Beyond 25 km, male spotted turtles were significantly less genetically similar than expected at 25–50 km and had a non‐significant trend to be less genetically similar at further distance classes (Table 5).

#### 3.3.2 Patterns in females

Spatial genetic structure of female *C. gutatta* significantly deviated from the null model (Figure 5, *ω* = 47.50, *p =* .001*, α* = 0.01). Females were significantly more likely to be genetically similar within shorter distance classes, 0.05–25 km, except from 0.5 to 1 km (Table 5). Beyond 25 km, female spotted turtles were less genetically similar than expected at 50–100 km and a non‐significant trend to be less genetically similar beyond 100 km (Figure 5; Table 5).

#### 3.3.3 Combined patterns of males and females

We found no significant differences between males and females (Figure 5, *ω* = 12.64, *p* = .557) We found no significant differences between males and females at any distance class using the T2 test (all *p* ≥ .202 except there was a trend for males to be less genetically similar from 25 to 50 km: *p* = .049, *α* = 0.01). This result was consistent with males and females showing similar patterns of dispersal, with no evidence for sex‐bias dispersal.

Because there were no significant differences in spatial genetic structure between males and females, we combined the sexes data to increase the spatial genetic structure resolution. Spatial genetic structure of the combined correlogram deviated from the null model (Figure 6, *ω* = 83.43, *p =* .0001). Individuals of *C. guttata* were more likely to be genetically similar at most shorter distance classes, 0–25 km (Figure 6, Table 5). Beyond 25 km, adult spotted turtles were significantly less genetically similar than random pairs of individuals in the study area (Figure 6; Table 5).

## 4 DISCUSSION

We found that both male and female *C. guttata*, separate and combined, had significant fine‐scale spatial genetic structure, with high relatedness at proximate locations <25 km and less than expected relatedness at distances beyond 25 km (Figures 3 & 5). We note that the largest distance between *C. guttata* to the maximum 25 km distance was 17 km and the smallest distance in the 25–50 km distance class was 30 km, so where in this 17–30 km range is too far for most *C. guttata* to travel is still to be determined. Little is known about dispersal distances in *C. guttata*, so knowledge that *C. guttata* rarely disperse to different sites beyond 17–30 km may be relevant to conservation of this species, as is determining if other landscape factors (e.g., large roads, rivers, and farmland) affect dispersal (Lamb, 2018).

Despite the detection of these strong patterns and restricted dispersal using genetic spatial autocorrelation (Peakall & Smouse, 2006; Smouse & Peakall, 1999), the sex‐specific patterns paralleled each other closely. There was, therefore, no evidence for sex‐biased dispersal (sensu Banks & Peakall, 2012). We had predicted we would detect male‐biased dispersal in *C. guttata* as it has been detected in almost all reptile species studied to date including turtles (e.g. Moore et al., 2020; Sheridan et al., 2010; but see Chapple & Keogh, 2005). Our expectations were due to male–male competition for mates without paternal investment in offspring being common in reptiles (Vitt & Caldwell, 2014) similar to mammals (Greenwood, 1980; Lawson Handley & Perrin, 2007). However, despite finding significant and strong patterns of fine‐scale genetic structure in our study in each sex, we detected no evidence for sex‐biased dispersal in *C. guttata* (Figures 3 & 5; Tables 4 & 5).

Our study contrasts with the handful of previous studies in turtles, which all detected male‐biased dispersal using population genetic methods. A single study inferred that a lack of female dispersal by nesting female *E. blandingii* may play a role in female philopatry by comparing nesting females to non‐nesting females and males combined, but that study did not compare dispersal between the two sexes (Reid et al., 2016). With the exception of that study, the other studies, which found male‐biased dispersal, were all conducted in saltwater turtles or fully terrestrial turtles, not freshwater turtles like *C. guttata*; two of these studies were in species in the same family as *C. guttata*, family Emydidae (Moore et al., 2020; Sheridan et al., 2010). However, in many reptile species, including turtles, males also have evolved, via sexual selection, larger size or more or brighter coloration compared to the females as a result of greater intensity of male–male interactions (reviewed in Brejcha & Kleisner, 2016). Unlike all the other reptile species tested for sex‐biased dispersal, *C. guttata* is unique because it exhibits sexual dimorphism in which the female has brighter coloration – not males, while males and females are of similar size (Figure 1; Ernst & Lovich, 2009; Rowe et al., 2012). The reasons for this reversal in sexual dimorphism are unknown, although plausibly it could be due to some degree of sex‐role reversal or female–female competition (sensu Blizzard & Pruett‐Jones, 2017). If females are competing to some degree, and male–male mating competition is reduced relative to females, sex biases in dispersal patterns can be altered (Le Galliard et al., 2006; Pérez‐González & Carranza, 2009). More research is needed to test these hypotheses in *C. guttata*. However, the potential lack of sex‐biased dispersal in *C. guttata* stands in stark contrast to other turtle species where male‐biased dispersal occurs (e.g., Moore et al., 2020; Sheridan et al., 2010, but see Chapple & Keogh, 2005). If there was a lack of male‐biased dispersal in any species where the ancestral pattern is male‐biased dispersal, it could be due to females having increased dispersal and/or males having reduced dispersal, both of which could change the ratio of male: female dispersal. The result of either of these behaviors would be dispersal patterns that are similar between the sexes (Figures 3 & 5) and not having the male‐biased dispersal present in other reptiles. However, despite significant spatial genetic structure being present for each sex hinting that sample sizes are adequate for identifying overall genetic spatial structure, it is still possible that a larger sample size could lead to detection of subtle differences between the sexes within distance classes (bins). More research is needed to determine if larger sample sizes are needed in this species compared to other similar studies in reptiles or if sex biased dispersal is present in this species in different geographic areas or different time periods (sensu Liebgold et al., 2013).

An additional seemingly minor, but methodologically important, result was that we found similar patterns whether or not we included a large number of microsatellite loci, relative to the total number of loci used (*n* = 5 out of *n* = 12), that were not in Hardy–Weinberg equilibrium or had heterozygote deficiency in a majority of populations (Table 3), potentially due to null alleles, to the dataset of loci that were in Hardy–Weinberg equilibrium in most populations (*n* = 7). In contrast to the assumption that loci deviating from Hardy–Weinberg equilibrium can cause Type I statistical errors, in our analyses, inclusion of the five loci did not change the results but simply increased resolution to a small degree, in a manner consistent with the increased resolution expected by the increased number of loci despite the likelihood that these loci each possessed reduced power. That the general, statistically significant, spatial genetic structure patterns for all turtles combined (Figure 4 vs. Figure 6) did not change, or became more significant, when we included loci that deviated from Hardy–Weinberg equilibrium supports the hypothesis that some factors, such as, potentially, null alleles may have weak effects on genetic analyses of spatial genetic structure (Chapuis & Estoup, 2007), at least in this one study. On the other hand, we cannot rule out that a lack of statistically significant sex‐biased dispersal in this study despite increased number of loci (Figure 3 vs. Figure 5) may stem from the additional loci (containing null alleles) providing weak additional power. As a result, it is possible that an overall number of loci studied that include loci with null alleles may provide a false sense of security that a sufficient number of loci have been utilized. Future research and modeling that compares the inclusion of loci that deviate from Hardy–Weinberg equilibrium is warranted to determine if using a test of Hardy–Weinberg equilibrium may or may not be appropriate litmus test to determine which loci should be included in certain types of population genetic analyses.

## 5 CONCLUSION

We did not detect differences between the sexes in fine‐scale spatial genetic structure that would have indicated sex‐biased dispersal occurs in *C. guttata*. However, we did detect significant overall fine‐scale spatial genetic structure in each sex and combined (Figures 4 & 6), with high relatedness in proximate locations <25 km and less than expected relatedness at distances beyond 25 km (Tables 4 & 5), indicating that the overall genetic patch size for *C. guttata* in this part of the Atlantic Coastal Plain is <25 km. This is important from the standpoint of conservation as rapid declines in *C. guttata*, due to factors such as habitat loss and the pet trade, have reduced the number of populations (Van Dijk, 2011). In our area, populations are typically 30–40 km apart (Lamb, 2018). Despite dispersal typically occurring within 25 km in our study between our nearest populations, it is still unclear if populations are becoming more genetically isolated. Genetic diversity remains high in *C. guttata* populations (Buchanan et al., 2019; this study). *Clemmys guttata* are long‐lived with a maximum lifespan between 51 and 110 years (Litzgus, 2006; Seburn, 2018), and most anthropogenic changes within a lifetime or two, so genetic tools may not be able to detect consequences of population isolation for at least several more generations (sensu McCluskey et al., 2016). Further studies should be conducted in this and other areas to identify if there are undiscovered proximate populations within 25 km of each other to determine if dispersal can alleviate the effects of potential future inbreeding depression (Buchanan et al., 2019) or if populations are currently too far apart for enough gene flow to alleviate inbreeding.

## AUTHOR CONTRIBUTIONS

**Eric B. Liebgold:** Conceptualization (equal); data curation (equal); formal analysis (equal); investigation (equal); methodology (equal); project administration (equal); resources (equal); software (equal); supervision (equal); validation (equal); visualization (equal); writing – original draft (equal); writing – review and editing (equal). **Myra J. Dickey:** Conceptualization (equal); formal analysis (equal); investigation (equal); methodology (equal); writing – original draft (equal); writing – review and editing (equal). **Stephanie M. Lamb:** Data curation (equal); investigation (equal); methodology (equal); writing – review and editing (equal). **Tami S. Ransom:** Data curation (equal); investigation (equal); methodology (equal); project administration (equal); writing – review and editing (equal). **Hunter Howell:** Data curation (supporting); investigation (supporting); writing – review and editing (supporting).

## Supporting information


Figure S1

## Data Availability

Microsatellite genotypes and associated data (sex, site number): https://doi.org/10.5061/dryad.w6m905qt6
