# Supplementary figures and images for: (Not) far from home: No sex bias in dispersal, but limited genetic patch size, in an endangered species, the Spotted Turtle (Clemmys guttata)
Source: Ecol Evol. 2023 Jan 4;13(1):e9734. doi: 10.1002/ece3.9734 (PMC9812832; doi:10.1002/ece3.9734)

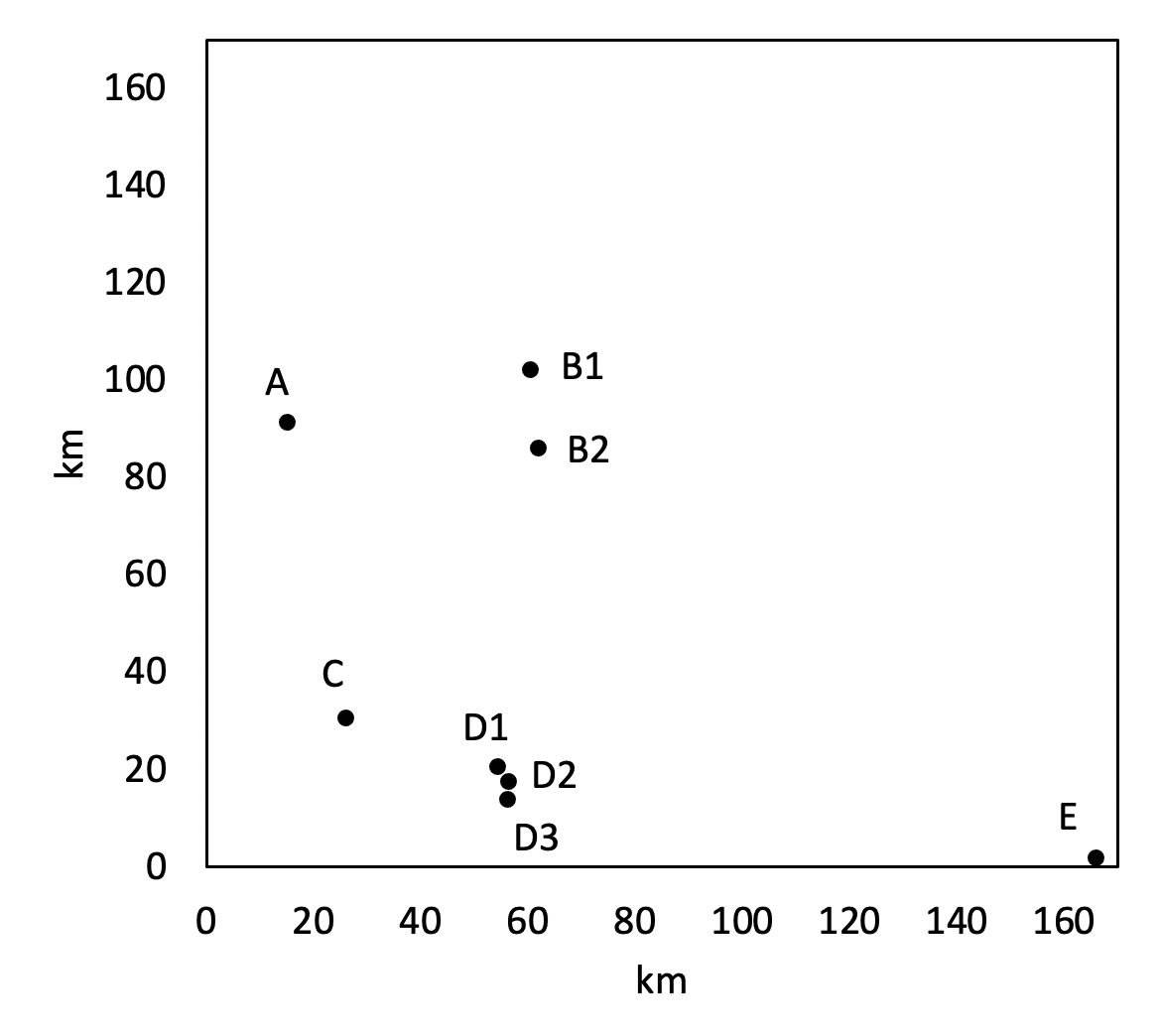

Supplement: Supplementary file 1 — Figure S1 [file ECE3-13-e9734-s001.zip › ECE3_9734_Supplemental Figure S1---.jpg]
